# Pharmacological treatment options for *mast cell activation disease*

**DOI:** 10.1007/s00210-016-1247-1

**Published:** 2016-04-30

**Authors:** Gerhard J. Molderings, Britta Haenisch, Stefan Brettner, Jürgen Homann, Markus Menzen, Franz Ludwig Dumoulin, Jens Panse, Joseph Butterfield, Lawrence B. Afrin

**Affiliations:** Institute of Human Genetics, University Hospital of Bonn, Sigmund-Freud-Strasse 25, 53127 Bonn, Germany; German Center for Neurodegenerative Diseases (DZNE), Bonn, Germany; Department of Oncology, Hematology and Palliative Care, Kreiskrankenhaus Waldbröl, Waldbröl, Germany; Allgemeine Innere Medizin, Gastroenterologie und Diabetologie, Gemeinschaftskrankenhaus, Bonn, Germany; Department of Hematology, Oncology and Stem Cell Transplantation, Medical Faculty, RWTH Aachen University, Aachen, Germany; Program for the Study of Mast Cell and Eosinophil Disorders, Mayo Clinic, Rochester, MN 55905 USA; Division of Hematology, Oncology, and Transplantation, University of Minnesota, Minneapolis, MN 55455 USA

**Keywords:** Mast cell, Mast cell activation disease, Systemic mastocytosis, Systemic mast cell activation syndrome, Therapy

## Abstract

*Mast cell activation disease* (MCAD) is a term referring to a heterogeneous group of disorders characterized by aberrant release of variable subsets of mast cell (MC) mediators together with accumulation of either morphologically altered and immunohistochemically identifiable mutated MCs due to MC proliferation (systemic mastocytosis [SM] and MC leukemia [MCL]) or morphologically ordinary MCs due to decreased apoptosis (MC activation syndrome [MCAS] and well-differentiated SM). Clinical signs and symptoms in MCAD vary depending on disease subtype and result from excessive mediator release by MCs and, in aggressive forms, from organ failure related to MC infiltration. In most cases, treatment of MCAD is directed primarily at controlling the symptoms associated with MC mediator release. In advanced forms, such as aggressive SM and MCL, agents targeting MC proliferation such as kinase inhibitors may be provided. Targeted therapies aimed at blocking mutant protein variants and/or downstream signaling pathways are currently being developed. Other targets, such as specific surface antigens expressed on neoplastic MCs, might be considered for the development of future therapies. Since clinicians are often underprepared to evaluate, diagnose, and effectively treat this clinically heterogeneous disease, we seek to familiarize clinicians with MCAD and review current and future treatment approaches.

## Introduction

**Fig. 1:**
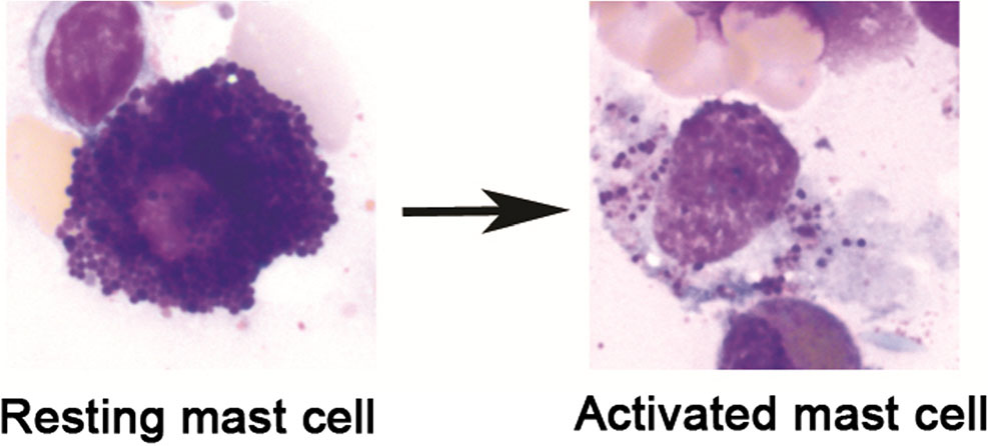
May-Grünwald/Giemsa stain of a resting human mast cell and a mast cell following activation-induced degranulation. Note the loss of granule staining. Mast cells obtained from the human bone marrow, magnification 1000×

Mast cells (MCs, Fig. 1) are immune cells of hematopoietic origin found in all human tissues, especially at the environmental interfaces. They act as both effector and regulatory cells and play a central role in adaptive and innate immunity (Anand et al. 2012; Gri et al. 2012). Their important role in immunological as well as non-immunological processes is reflected by the large number of mediators (>200) including pre-stored ones such as histamine and tryptase as well as numerous mediators synthesized de novo in response to allergic or non-immune triggers such as chemokines and cytokines, by which MCs may influence other cells (Lundequist and Pejler 2011; Ibelgaufts 2016). Their evolved arrays of sensory and response mechanisms engender diverse havoc when MC dysfunction emerges.

The umbrella term *mast cell activation disease* (MCAD; Akin et al. 2010) comprises the full spectrum of primary systemic MC disease, i.e., *systemic mastocytosis* (SM) which is further divided into several subtypes (Valent et al. 2007; Tables 1 and 2), primary *MC activation syndrome* (MCAS; Table 3; Molderings et al. 2011a; Hamilton et al. 2011; Valent et al. 2012), and *MC leukemia* (MCL). Pathogenetically, MCAD denotes a group of polygenic MC disorders (Molderings 2015, 2016) characterized by aberrant release of variable subsets of MC mediators and also an accumulation of either morphologically altered and immunohistochemically identifiable mutated MCs due to MC proliferation (SM and MCL) or morphologically ordinary MCs due to decreased apoptosis (MCAS; Kohno et al. 2005; Aichberger et al. 2009; Karlberg et al. 2010a). According to recent molecular genetic findings (Molderings 2015, 2016; Haenisch et al. 2014; Lasho et al. 2016), the subclasses and clinical subtypes of MCAD do not represent distinct disease entities but should be more accurately regarded as variable presentations of a common generic state of MC dysfunction (Molderings et al. 2007, 2010; Hermine et al. 2008; Akin et al. 2010). Due to both the widespread distribution of MCs and the great heterogeneity of aberrant mediator expression patterns, symptoms can occur in virtually all organs and tissues; hence, the clinical presentation of MCAD is very diverse, sometimes to the even-further-confounding point of presenting opposite abnormalities in different patients (or even in the same patient at different times, or in different sites in the same patient at the same time). While the prevalence of SM in Europeans ranges between 0.3 and 13 per 100,000 (Haenisch et al. 2012; Cohen et al. 2014; van Doormaal et al. 2013), the prevalence of MCAS may be as high as 17 % (in Germany; Molderings et al. 2013a, b).

**Table 1:** WHO 2008 diagnostic criteria for systemic mastocytosis (Valent et al. 2001)

Major criterion:
1. Multifocal, dense aggregates of MCs (15 or more) in sections of the bone marrow or other extracutaneous tissues and confirmed by tryptase immunohistochemistry or other special stains Minor criteria: 1. Atypical or spindled appearance of at least 25 % of the MCs in the diagnostic biopsy 2. Expression of CD2 and/or CD25 by MCs in the marrow, blood, or extracutaneous organs 3. KIT codon 816 mutation in the marrow, blood, or extracutaneous organs 4. Persistent elevation of serum total tryptase >20 ng/ml

Diagnosis of SM made by either (1) the major criterion plus any one of the minor criteria or (2) any three minor criteria

**Table 2:** Classification of systemic mastocytosis (modified form Valent et al. 2007)

Categories of systemic mastocytosis (SM)	Subtypes
Indolent systemic mastocytosis	• Smoldering systemic mastocytosis • Isolated bone marrow mastocytosis • Well-differentiated systemic mastocytosis
Aggressive systemic mastocytosis (ASM)	• ASM in transformation
Systemic mastocytosis with an associated clonal hematological non-mast cell lineage disease	• SM-acute myeloid leukemia • SM-myelodysplastic syndrome • SM-myeloproliferative neoplasm • SM-chronic myelomonocytic leukemia • SM-chronic eosinophilic leukemia • SM-non-Hodgkin lymphoma • SM-multiple myeloma

**Table 3:** Current provisional criteria to define *mast cell activation syndrome* (MCAS; modified from Afrin and Molderings 2014)

Major criterion		
	Constellation of clinical complaints attributable to pathologically increased mast cell activity (mast cell mediator release syndrome)	
Minor criteria		
1.	Focal or disseminated increased number of mast cells in marrow and/or extracutaneous organ(s) (e.g., gastrointestinal tract biopsies; CD117-, tryptase-, and CD25-stained)	
2.	Abnormal spindle-shaped morphology in >25 % of mast cells in marrow or other extracutaneous organ(s)	
3.	Abnormal mast cell expression of CD2 and/or CD25 (i.e., co-expression of CD117/CD25 or CD117/CD2)	
4.	Detection of genetic changes in mast cells from the blood, bone marrow, or extracutaneous organs for which an impact on the state of activity of affected mast cells in terms of an increased activity has been proven	
5.	Evidence (typically from body fluids such as whole blood, serum, plasma, or urine) of above-normal levels of mast cell mediators including:	
	•	Tryptase in the blood
	•	Histamine or its metabolites (e.g., *N*-methylhistamine) in the urine
	•	Heparin in the blood
	•	Chromogranin A in the blood (potential confounders of cardiac or renal failure, neuroendocrine tumors, or recent proton pump inhibitor use were excluded)
	•	Other relatively mast cell-specific mediators (e.g., eicosanoids including prostaglandin PGD_2_, its metabolite 11-β-PGF_2α_, or leukotriene E4)
6.	Symptomatic response to inhibitors of mast cell activation or mast cell mediator production or action (e.g., histamine H_1_ and/or H_2_ receptor antagonists, cromolyn)	

Diagnosis of MCAS made by either (1) the major criterion plus any one of the minor criteria or (2) any three minor criteria

This review focuses on the current state of drug therapy in SM and MCAS and describes perspectives of promising new approaches for drug treatment. Compounds in various stages of preclinical and clinical development are summarized in tables. We first describe drugs that are currently available and either are used on a regular basis in MCAD therapy or have been used successfully in single MCAD cases. In this context, it should be noted that there is no official guideline for treatment of MCAD.

## Treatment options

**Fig. 2:**
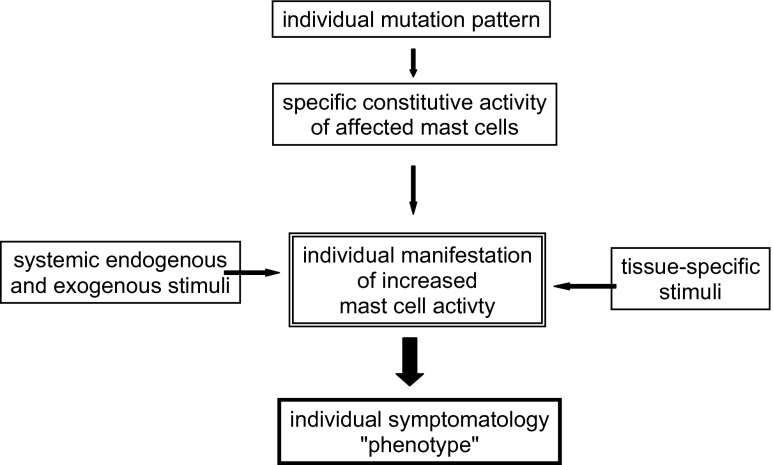
Scheme of conditions responsible in MCAD for the development of individual phenotypes

Due to its genetic roots, MCAD generally is regarded as incurable. Recent mutational studies revealed that each patient has an individual pattern of genetic and epigenetic alterations which may affect the intracellular signal transduction pathways and receptive sites involved in sensory perception. As a consequence, mediator formation and release as well as inhibition of apoptosis and/or increase in proliferation are determined by individual genetic and epigenetic conditions (Fig. 2) and represent potential targets for therapy. Hence, there is need of highly personalized therapy for the disease. Unfortunately (with regard to easy detection), most genetic alterations (with a few exceptions such as certain mutations in tyrosine kinase KIT, e.g., KIT^D816V^) do not alter the morphology and immunohistochemistry of the surface of the affected MCs. Thus, in most cases except for patients with the reliably identifiable D816V mutation, it cannot be decided by simple tests whether MCs found in biopsies are genetically altered MCs or physiological MCs.

### First-line treatment options

Step 1 in managing most situations of inappropriate MC activation is identifying the individual patient’s unique triggers (chemical, physical, or otherwise) as precisely as possible and then desensitizing when possible (in truth, rarely) and otherwise practicing avoidance. With respect to drug treatment, only a few clinical therapeutic trials have been conducted in SM (midostaurin, cladribine, masitinib; Table 4), and there have been no therapeutic trials in MCAS yet. Most information about therapeutic effectiveness in MCAD has been found in small case series (Table 4) and single case reports, perhaps unsurprising given the mutational heterogeneity of the disease and thus the heterogeneity of its patterns of clinical presentation and therapeutic responsiveness. Therefore, in the future, it may be helpful to establish an international patient registry in partnership with existing registries so that issues related to molecular and clinical MCAD phenotypes can be adequately addressed. As the primary feature of MCAD is inappropriate MC activation (Molderings et al. 2011a, b; Pardanani 2013; Cardet et al. 2013), mainstays of first-line management are identification and avoidance of triggers plus therapies to control MC mediator production (both primary as well as secondary/reactive; Table 5) as well as their action (Table 6).

**Table 4:** Case series and clinical therapeutic trials in systemic mastocytosis and mast cell activation syndrome

Compound	Number of patients included in the study or case series	References
H_1_-antihistamines		
Rupatadine	30	Siebenhaar et al. 2013
Azelastine vs. chlorpheniramine	15	Friedman et al. 1993
Ketotifen vs. hydroxyzine	8	Kettelhut et al. 1989
Chlorpheniramine plus cimetidine	8	Frieri et al. 1985
Continuous diphenhydramine infusion	10	Afrin 2015 ^a^
Mast cell stabilizer		
Cromoglicic acid (cromolyn)	5	Soter et al. 1979
	11	Horan et al. 1990
	4	Mallet et al. 1989
	8	Frieri et al. 1985
	2	Welch et al. 1983
	2	Zachariae et al. 1981
Tranilast	2	Katoh et al. 1996
Kinase inhibitors		
Imatinib (STI571)	14	Droogendijk et al. 2006
	20	Vega-Ruiz et al. 2009
	22	Lim et al. 2009
	17	Pagano et al. 2008
	12	Pardanani et al. 2003
	5	Heinrich et al. 2008
	3	Hennessy et al. 2004
Nilotinib (AMN107)	61	Hochhaus et al. 2015
Dasatinib (BMS-354825)	33	Verstovsek et al. 2008
	4	Purtill et al. 2008
Midostaurin (PKC412)	9	Papayannidis et al. 2014
	11	Knapper et al. 2011
	22	Chandesris et al. 2014
	89	Gotlib et al. 2014
	14	Strati et al. 2015
Masitinib	25	Paul et al. 2010
Cytostatic agents		
Hydroxyurea	26	Lim et al. 2009
	5	Afrin 2013 ^a^
Cladribine (2-chlorodeoxyadenosine)	22	Lim et al. 2009
	10	Kluin-Nelemans et al. 2003
	4	Pardanani et al. 2004
	3	Pagano et al. 2008
	68	Barete et al. 2015
Immunomodulation		
Interferon-α	20	Casassus et al. 2002
	5	Hauswirth et al. 2004
	10	Laroche et al. 2011
	40	Lim et al. 2009
	8	Pagano et al. 2008
	6	Giraldo Castellano et al. 1998
	9	Hennessy et al. 2004
	3	Worobec et al. 1996
Thalidomide	16	Gruson et al. 2013
IgE antibody		
Omalizumab	4	Molderings et al. 2011b ^a^
	2	Carter et al. 2007
	2	Lieberoth and Thomsen 2015
ß-Sympathomimetics		
Isoprenaline, terbutaline	5	van Doormaal et al. 1986
Cyclooxygenase inhibitor		
Acetylsalicylic acid	4	Butterfield and Weiler 2008
	20	Butterfield 2009

^a^It indicates clinical trials performed with patients with mast cell activation syndrome

**Table 5:** First-line drugs which can potentially be used in the treatment of mast cell (MC) activation disease and their target location and mechanisms of action

	Target location/mechanisms of action	Growth inhibition	Decrease of mediator release	To relieve symptoms	References
First-line drugs					
H_1_-antihistamines (preferably of the second and third generations)	Block mutual activation of mast cells via H_1_-histamine receptors; antagonize H_1_-histamine receptor-mediated symptoms		X	X	Church and Gradidge 1980 Valent et al. 2007R Picard et al. 2013R Nurmatov et al. 2015 Siebenhaar et al. 2013 Escribano et al. 2006R
H_2_-antihistamines	Block mutual activation of mast cells via H_2_-histamine receptors; antagonize H_2_-histamine receptor-mediated symptoms		X	X	Valent et al. 2007R Escribano et al. 2006R
Cromoglicic acid (also known as cromolyn)	GPR35; modulation of chloride current		X	X	Soter et al. 1979 Valent et al. 2007R Yang et al. 2010 Edwards et al. 2011 Edwards and Hagberg 2010 Zhang et al. 2016 Escribano et al. 2006R
Vitamin C	Increased degradation of histamine; decrease of histamine formation by inhibition of histidine decarboxylase		X	X	Hagel et al. 2013 Johnston et al. 1992 Uchida et al. 1989 Chatterjee et al. 1975

As a rule, these drugs should be used in combination to achieve a sufficient reduction of MC activity. All drugs should be tested for tolerance in a low single dose before therapeutic use, if their tolerance in the patient is not known from an earlier application. A precondition for therapeutic success is the avoidance of identifiable triggers of MC activation; in this context, parallel to the beginning of drug therapy, gluten, cow milk protein, and baker’s yeast should be omitted from the diet for 3–4 weeks

*R* review article (further references therein)

**Table 6:** Symptomatic treatment (orally as needed) in MCAD (modified from Molderings et al. 2014)

Colitis ⇒ budesonide; for some days, prednisone >20 mg/day
Diarrhea ⇒ c(h)olestyramine; nystatin; montelukast; 5-HT_3_ receptor inhibitors (e.g. ondansetron); incremental doses of acetylsalicylic acid (50–350 mg/day; extreme caution because of the possibility to induce mast cell degranulation); in steps test each drug for 5 days until improvement of diarrhea
Colicky abdominal pain due to distinct meteorism ⇒ metamizole; butylscopolamine
Angioedema ⇒ tranexamic acid; icatibant
Nausea ⇒ dimenhydrinate; lorazepam; 5-HT_3_ receptor inhibitors; NK1 antagonists such as aprepitant
Respiratory symptoms (mainly due to increased production of viscous mucus and obstruction with compulsive throat clearing) ⇒ leukotriene receptor blockers such as montelukast; if in a country available, leukotriene synthesis inhibitors such as zileuton; urgent: short-acting ß-sympathomimetic
Gastric complaints ⇒ proton-pump inhibitors (de-escalating dose-finding)
Osteoporosis, osteolysis, bone pain ⇒ bisphosphonates (vitamin D plus calcium application is second-line treatment in MCAD patients because of limited reported success and an increased risk for developing kidney and ureter stones); calcitonin; teriparatide (with caution; cases of cholestatic liver failure due to this drug have been reported); anti-RANKL drugs such as denosumab (dental clearance is required prior to treatment with bisphosphonates and anti-RANKL therapies due to risk for potentially severely morbid osteonecrosis of the jaw in patients with poor dentition or recent invasive dental work)
Non-cardiac chest pain ⇒ when needed, additional dose of a H_2_-histamine receptor antagonist; also, proton-pump inhibitors for proven gastroesophageal reflux
Tachycardia ⇒ AT_1_-receptor antagonists; ivabradine
Neuropathic pain and paresthesia ⇒ α-lipoic acid
Itches ⇒ palmitoylethanolamine-containing care products; cromolyn-containing ointment
Rheumatoid symptoms ⇒ COX2 inhibitors such as etoricoxib or celecoxib; paracetamol
Anemia ⇒ in iron-deficiency anemia, iron supplementation (whether oral or parenteral) must be given cautiously due to risk for potentially intense mast cell activation; alternatively, red blood cell transfusion should be considered
Interstitial cystitis ⇒ pentosan, amphetamines
Sleep-onset insomnia/sleep-maintenance insomnia ⇒ triazolam
Conjunctivitis ⇒ exclusion of a secondary disease; otherwise preservative-free eye drops with H_1_-antihistamine, cromolyn, ketotifen, or glucocorticoid for brief courses
Hypercholesterolemia ⇒ (probably due to inhibition of transport into the cells, thus independent of diet) >300 mg/dL therapeutic trial with HMG-CoA reductase inhibitor atorvastatin

### Subordinate therapeutic options

#### Continuous diphenhydramine infusion

Occasional patients suffer nearly continuous anaphylactoid and/or dysautonomic states poorly controlled by intermittently dosed epinephrine, antihistamines, and steroids. As discussed in more detail below, some such patients are particularly triggered by a wide range of medication excipients, making it challenging for them to tolerate trials of any adulterated (non-pure) medications, and yet some modicum of stability is required to pursue medication trials in such patients. Diphenhydramine is a well-tolerated histamine H_1_ receptor blocker (that among other non-threatening adverse affects can cause dizziness and an increase in appetite) which can quickly suppress MC activation and is used to treat allergic reactions and anaphylaxis. However, its half-life is as short as 1 h (www.drugbank.ca/drugs/DB01075). Intermittently dosed, though, its initial therapeutic serum level rapidly declines to subtherapeutic levels and the patient seesaws into yet another flare. The safety of continuous diphenhydramine infusion was established in trials of the “BAD” regimen (diphenhydramine [Benadryl], lorazepam [Ativan], and dexamethasone) in refractory chemotherapy-induced emesis in adult and pediatric patients (Dix et al. 1999; Jones et al. 2007). In a small series of ten MCAS patients suffering almost continuous anaphylactoid/dysautonomic flares, continuous diphenhydramine infusion at 10–14.5 mg/h appeared effective in most patients at dramatically reducing flare rates and appeared safely sustainable at stable dosing for at least 21 months (Afrin 2015). Stabilization has enabled successful trials of other helpful medications, but no patient has yet successfully stopped continuous diphenhydramine infusion.

#### Acute and chronic immunosuppressive therapies

**Fig. 3:**
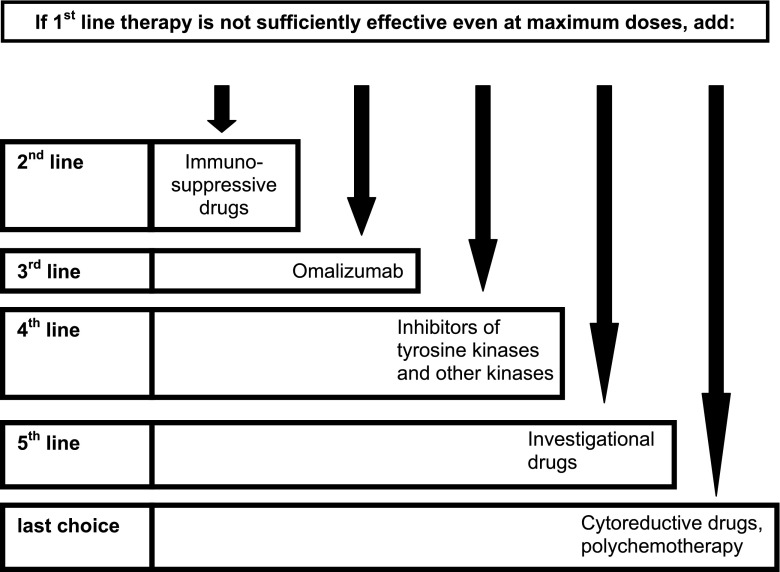
Suggested treatment options for mast cell activation disease. All drugs should be tested for tolerance in a low single dose before therapeutic use, if their tolerance in the patient is not known from an earlier application. For further details of indication, see text

Though typically not first-line, acute and chronic immunosuppressive therapies can be considered (Fig. 3; Table 7) and may be particularly appropriate for patients possibly manifesting an autoimmune component of the disease as might be suggested by the presence, for example, of anti-IgE or anti-IgE-receptor antibodies. Glucocorticoids may exert beneficial effects in MCAD, including a decrease in production of stem cell factor (SCF, and possibly other cytokines) and a decrease in MC activation, by various mechanisms which have been extensively reviewed by Oppong et al. 2013. Glucocorticoids at doses >20 mg prednisone equivalent per day are frequently needed to effectively control otherwise refractory acute (and chronic) symptoms. Their chronic toxicity profile is disadvantageous for long-term use, but such toxicities have to be accepted in some cases. The influence of azathioprine, methotrexate, ciclosporine, hydroxyurea, and tamoxifen on MC activity can vary from no to moderate effect depending on individual disease factors. As in therapy of rheumatoid arthritis, azathioprine and methotrexate can be used in daily doses lower than those used in cancer or immunosuppressive post-transplant therapy. Effective MCAD therapy with ciclosporine requires doses as high as those used in transplantation medicine (M. Raithel, personal communication). Methotrexate has to be administered parenterally to be effective (unpublished observation, G.J. Molderings), and in the risk-benefit analysis, a possible non-immunologic histamine release from MCs (Estévez et al. 1996) has to be considered. Hence, use of the compound should be limited to MCAD with methotrexate-sensitive comorbidities (e.g., rheumatoid arthritis and vasculitis).

**Table 7:** Second- and third-line drugs which can potentially be used in the treatment of mast cell activation disease and their target location and mechanisms of action

	Target location/mechanisms of action	Growth inhibition	Decrease of mediator release	To relieve symptoms	References
Second-line drugs	Immunosuppressive drugs				
Azathioprine	Multiple targets		X	X	Nolte and Stahl Skov 1988, Own unpublished data
Ciclosporine	Calcineurin inhibitor		X	X	Kurosawa et al. 1999, Broyd et al. 2005, Trojan and Khan 2012, Own unpublished data
Glucocorticoids	Multiple targets	(X)	X	X	Zen et al. 2011R
Hydroxyurea	Multiple targets	X		X	Lim et al. 2009, Afrin 2013
Tamoxifen	Precise mechanism of action in MCAD unknown	X	X	In single cases	Butterfield and Chen 2016, Duffy et al. 2003;
Methotrexate	Multiple targets		?	X	Sagi et al. 2011, Vrugt et al. 2000
Third-line drugs					
Omalizumab	Anti-IgE antibody			X	Molderings et al. 2011b Bell and Jackson 2012; Kibsgaard et al. 2014 Kontou-Fili et al. 2010
Etoricoxib Acetylsalicylic acid	COX-inhibitors			X	Butterfield and Weiler 2008 Breslow et al. 2009 Butterfield 2009
Montelukast	Antagonist at cys-LT_1_ receptors			X	Tolar et al. 2004 Cikler et al. 2009 Breslow et al. 2009 Turner et al. 2012
Zileuton	5-Lipoxygenase inhibitor			X	Rodriguez et al. 2011

*R* review article (further references therein)

Recently, the humanized anti-IgE murine monoclonal antibody omalizumab has been described in multiple case reports as safe and effective in MCAD (e.g., Molderings et al. 2011b; Kontou-Fili et al. 2010; Bell and Jackson 2012; Kibsgaard et al. 2014), though a definitive trial has yet to be conducted. Since treatment with omalizumab has an acceptable risk-benefit profile, it should be considered in cases of MCAD resistant to at least a few lines of therapy. The drug’s expense likely consigns it to third-line (or later) treatment (Table 7). If elevated prostaglandin levels induce symptoms such as persistent flushing, inhibition of cyclooxygenases by incremental doses of acetylsalicylic acid (ASA; 50–350 mg/day) may be used with extreme caution, since ASA can induce MC degranulation probably due its chemical property as an organic acid. The leukotriene antagonist montelukast (possibly more effective at twice-daily dosing; personal observation, L.B. Afrin) and the 5-lipoxygenase inhibitor zileuton may be useful adjuvants in people with MCAD, particularly in those with refractory gastrointestinal and urinary symptoms (Tolar et al. 2004; Turner et al. 2012; Akhavein et al. 2012).

Studies of kinase inhibitors, both on-market (e.g., imatinib, nilotinib, dasatinib) and experimental (e.g., midostaurin, masitinib), have yielded variable responses in SM ranging from no response to partial or even complete responses (Fig. 3; Table 8). As with all drugs used in therapy of MCAD, their therapeutic success seems to be strongly dependent on the individual patient, again underscoring the observed mutational heterogeneity of the disease. In formal studies in SM patients, although some kinase inhibitors reduced MC burden as reflected by histological normalization in bone marrow and improved laboratory surrogate markers (e.g., tryptase level in blood), at best only partial improvement of mediator-related symptoms was achieved (Droogendijk et al. 2006; Gotlib et al. 2008; Verstovsek et al. 2008; Vega-Ruiz et al. 2009). There has been repeated suggestion that symptoms in MCAD may be due more to mediator release from normal MCs secondarily activated by pathologically overactive, mutated MCs (Galli and Costa 1995; Rosen and Goetzl 2005; Boyce 2007; Kaneko et al. 2009; Fig. 2 in Molderings et al. 2014), helping to explain why intensity and pattern of symptoms do not correlate with degree of MC proliferation and infiltration (Topar et al. 1998; Hermine et al. 2008; Broesby-Olsen et al. 2013; Erben et al. 2014; Quintás-Cardama et al. 2013). Distinction in pathways in the MC which promote MC proliferation vs. mediator production/release may explain why kinase inhibitors reduce MC burdens and MC-driven symptoms to different degrees (Droogendijk et al. 2006; Gotlib et al. 2008; Verstovsek et al. 2008; Vega-Ruiz et al. 2009; Table 8). However, in some case reports, kinase inhibitors have been significantly effective at relieving symptoms. Thus, in spite of potential serious adverse effects of these drugs, a therapeutic trial may be justified in individual cases at an early stage. Partial and complete responses have been reported with some of these agents in MCAS too (e.g., Afrin 2010, 2011, 2012, 2015; Afrin et al. 2015a). Dosing of the kinase inhibitors in the individual often is considerably lower than how such drugs are dosed for other applications (e.g., imatinib, sunitinib; Afrin et al. 2015a). Possibly due to the causative mutations in multiple genes leading to simultaneous activation of multiple intracellular pathways, multitargeted kinase inhibitors such as midostaurin and sunitinib may be more effective than drugs which selectively downregulate only one intracellular pathway.

**Table 8:** Kinase inhibitors which can potentially be used as fourth-line drugs in the treatment of mast cell activation disease and their target location and mechanisms of action

	Target location/mechanisms of action	Growth inhibition	Decrease of mediator release	To relieve symptoms	References
Fourth-line drugs	Inhibitors of tyrosine kinases and other kinases				
Imatinib	KIT (excluding D816X), PDGFR, Bcr-Abl, Arg/Abl2, DDR-1	X	(X)	X	Pardanani et al. 2003 Droogendijk et al. 2006 Lim et al. 2009 Vega-Ruiz et al. 2009 Aman et al. 2012 Vaali et al. 2012 Quintás-Cardama et al. 2011R Marton et al. 2015
Nilotinib	KIT, PDGFR, Bcr-Abl	X		(X)	Hochhaus et al. 2006 Quintás-Cardama et al. 2011R Hochhaus et al. 2015 El-Agamy 2012
Dasatinib	KIT, BCR-ABL1, Lyn, Btk, Tec	X		(X)	Verstovsek et al. 2008 Hantschel et al. 2007 Gleixner et al. 2011 Quintás-Cardama et al. 2011R
Sunitinib	VEGFR, PDGFR, KIT, FLT3, RET, CSF1R, SRC, 313 potential kinase targets	X	X	X	Afrin et al. 2015a Yamaki and Yoshino 2012 Papaetis and Syrigos 2009 Bairlein 2010
Masitinib	KIT, PDGFRα, Lck, LYN, FGFR3, FAK	X		X	Marech et al. 2014 Moussy and Kinet 2014 Paul et al. 2010 Quintás-Cardama et al. 2011R
Midostaurin	PKC, FLT3, KIT, PDGFR, VEGFR2	X	X	X	Gotlib et al. 2014 Papayannidis et al. 2014 Knapper et al. 2011 Quintás-Cardama et al. 2011R
Ponatinib	Bcr-Abl, KIT, FLT3, FGFR1, PDGFRα, Lyn	X			Jin et al. 2014 Gleixner et al. 2013
Bafetinib	KIT (excluding D816X), Abl, Lyn	X			Peter et al. 2010a
Bosutinib	Lyn, Btk	X		In ASM patients ineffective	Gleixner et al. 2011 Randall et al. 2015

*R* review article (further references therein)

In the mastocytosis patient with significant MC burden and/or an aggressive clinical course, cytoreductive drugs are prescribed (Lim et al. 2009; Valent et al. 2010). Unfortunately, effective cytoreductive therapies in SM presently are few in number and typically offer only modest response rates, qualities, and durations. Cytoreductive options include interferon-α and 2-chlorodeoxyadenosine (cladribine, 2-CdA; Fig. 3 and Table 9). Interferon-α is frequently combined with prednisone and is commonly used as cytoreductive therapy for aggressive SM. It ameliorates mastocytosis-related organopathy in a proportion of cases but can be associated with considerable adverse effects (e.g., flu-like symptoms, myelosuppression, depression, hypothyroidism), which may limit its use in MCAD (Simon et al. 2004; Butterfield 2005). PEGylated interferon-α has been shown to be as efficacious as and less toxic than the non-PEGylated form in some myeloproliferative neoplasms, but it has not been specifically studied in MCAD. 2-Chlorodeoxyadenosine is generally reserved for last-choice treatment of patients with aggressive SM who are either refractory or intolerant to interferon-α. Potential toxicities of 2-CdA include significant and potentially prolonged myelosuppression and lymphopenia with increased risk for opportunistic infections.

**Table 9:** Last-choice drugs which can potentially be used in the treatment of mast cell activation disease and their target location and mechanisms of action. R-review article (further references therein)

	Target location/mechanisms of action	Growth inhibition	Decrease of mediator release	To relieve symptoms	References
Last-choice drugs					
Interferon-α	Multiple targets	X		(X)	Simon et al. 2004 Casassus et al. 2002 Hauswirth et al. 2004 Butterfield et al. 2005 Butterfield 2005R Yoshida et al. 2009 Lim et al. 2009 Quintás-Cardama et al. 2011R
Cladribine	Nucleoside analog	X	X	X	Tefferi et al. 2001 Kluin-Nelemans et al. 2003 Pardanani et al. 2004 Lim et al. 2009 Böhm et al. 2010 Radojković et al. 2011 Quintás-Cardama et al. 2011R Lock et al. 2015 Barete et al. 2015

#### Last resorts

Polychemotherapy, including intensive induction regimens of the kind used in treating acute myeloid leukemia, as well as high-dose therapy with stem cell rescue, are approaches restricted to rare, selected patients. Allogeneic stem cell transplantation sometimes yields remissions in mastocytosis long thought impermanent (Spyridonidis et al. 2004; Nakamura et al. 2006; Bae et al. 2013; Gromke et al. 2013), though recent data may offer new hope (Ustun et al. 2014).

### Investigational drugs

There are several drugs approved for indications other than MCAD which already have been successfully used in isolated cases with MCAD (Table 10). In cases of unsuccessful first- to fourth-line therapy, these compounds may be considered as treatment options.

**Table 10:** Drugs successfully (or not) used off-label to treat isolated cases of mast cell activation disease

	Target location/mechanisms of action	Growth inhibition	Decrease of mediator release	To relieve symptoms	References
Investigational drugs					
Thalidomide	Precise mechanism of action unknown			X	Damaj et al. 2008 Gruson et al. 2013
Lenalidomide				No effect	Kluin-Nelemans et al. 2009
Flavonoids (e.g., luteolin, quercetin, genistein)	Multiple	X	(X)	(X)	Alexandrakis et al. 2003 Kempuraj et al. 2006 Min et al. 2007 Finn and Walsh 2013R Weng et al. 2012 Lee et al. 2015 Weng et al. 2015
Miltefosine	Raft modulator		X	(X)	Weller et al. 2009 Maurer et al. 2013R
Mepolizumab	IL-5 antibody	X			Otani et al. 2012
Rituximab	CD20 antibody			X	Borzutzky et al. 2014
Ruxolitinib	JAK	X		X	Yacoub and Prochaska 2016 Kvasnicka et al. 2014
Cannabinoids	Agonists at the cannabinoid receptors			X	De Filippis et al. 2008 Frenkel et al. 2015 Own unpublished experiences
Methylene blue	Guanylyl cyclase inhibitor			Anaphylaxis treatment	Rodrigues et al. 2007 Evora and Simon 2007R
Pimecrolimus	Calcineurin inhibitor	X		Cutaneous symptoms; (mice)	Ma et al. 2010 Correia et al. 2010
Everolimus	mTOR			no effect	Parikh et al. 2010
Ribavirin	Possibly suppression of activated retroviral elements in the genome which may be involved in the development of the somatic mutations in KIT and other proteins		X	X	Marquardt et al. 1987 Molderings 2016 Own unpublished experiences

*R* review article (further references therein)

A variety of drugs have been shown to inhibit MC growth, to decrease MC mediator release, and/or to relieve mediator-induced symptoms in in vitro and in vivo animal models (Table 11). Some of these drugs are approved for certain indications (such as ambroxol, statins, mefloquine, and ruxolitinib) and, thus, may be used (if accessible given financial considerations for some agents) if MCAD patients suffer from both the disorder of indication (e.g., hypercholesterolemia—statins, mucous congestion—ambroxol, polycythemia vera—ruxolitinib) and MCAD. An important question is what the role of the other compounds without approved indications should be in clinical practice. There are several challenges that may hamper the clinical introduction of novel targeted therapies in general. Some of these challenges include inherent problems in the translation of preclinical findings to the clinic, the presence of multiple coactive deregulated pathways in the disease, and questions related to the optimal design of clinical trials (e.g., eligibility criteria and endpoints). In particular, the testing of novel targeted treatment in an isolated fashion may be problematic and may in fact underestimate the effectiveness of these novel compounds. It is reasonable to assume that combination therapy will be the key to target parallel critical pathways.

**Table 11:** Investigational drugs which might have activity against mast cell activation disease since they induce apoptosis of mast cells and/or suppress mast cell mediator release in vitro and/or in vivo

	Target location/mechanisms of action	Growth inhibition	Decrease of mediator release	To relieve symptoms	Investigated in vitro	Investigated in vivo	References
Investigational drugs							
ABT-737 {(*R*)-4-(3-dimethylamino-1-phenylsulfanylmethyl-propylamino)-*N*-{4-[4-(4′-chloro-biphenyl-2-ylmethyl)-piperazin-1-yl]-benzoyl}-3-nitro-benzenesulfonamide)}	BH3 mimetic	X			Murine BMMC, human cord blood-derived MCs, C57 MC line, MC/9 MC line	Mice	Karlberg et al. 2010b
17-Allylamino-17-demethoxygeldanamycin, Ganetespib (STA-9090)	Binding to heat shock protein 90	X			HMC-1, canine BMMC, C2 MC line, BR canine mastocytoma cell lines		Fumo et al. 2004 Lin et al. 2008
Ambroxol	Multiple		X		Human MCs		Gibbs et al. 1999
Amitriptyline, clomipramine, maprotiline	Yet to be defined in MCAD		X			Male Wistar rats	Gurgel et al. 2013 Clemons et al. 2011
Benzodiazepines	Yet to be defined	(X)	X	X			Molderings et al. 2013b; Dueñas-Laita et al. 2009; Bidri et al. 1999; Fujimoto et al. 2005; Suzuki-Nishimura et al. 1989; Hoffmann et al. 2013
BI 2536 {(*R*)-4-(8-cyclopentyl-7-ethyl-5-methyl-6-oxo-5,6,7,8-tetrahydropteridin-2-ylamino)-3-methoxy-*N*-(1-methylpiperidin-4-yl)benzamide}	Polo-like kinase-1	X			HMC-1, primary human neoplastic MCs		Peter et al. 2011
BLU-285 (chemical structure not yet published)	KIT	X			HMC-1.2, P815 mouse mastosarcoma cells		Evans et al. 2015
Botulinum toxin A	Cleavage of the SNARE proteins	X	X			SD rats	Park 2013
Butaprost	EP_2_ receptor agonist		X		Human lung MCs		Kay et al. 2006
Cerivastatin, fluvastatin, atorvastatin	Unknown in MCAD	X	X		Primary human MCs, HMC-1, P815		Krauth et al. 2006 Paez et al. 2015
Chemokine receptor antagonists	Targeting activating chemokine receptors expressed on MCs		X			Mice	Koelink et al. 2012R
Cinnamaldehyde	Signaling molecules, e.g., ERK1/2, JNK, p38, Akt		X		Human MCs, RBL-2H3 cells		Hagenlocher et al. 2015 Bibi et al. 2014R
Combined arginine and glutamine	Multiple		X		Human intestinal MCs		Lechowski et al. 2013
Coumarines (scopoletin)	Yet to be defined in MCAD		X		HMC-1		Moon et al. 2007 Finn and Walsh 2013R
CRA1000 {*N-*ethyl-4-[4-(3-fluorophenyl)-3,6-dihydro-2*H*-pyridin-1-yl]-6-methyl-*N*-(2-methylsulfanyl-4-propan-2-ylphenyl)pyrimidin-2-amine}	Non-peptidic corticotropin-releasing factor antagonist		X			Mouse dermal MCs	Shimoda et al. 2010
Crenolanib	FLT3	X			HMC-1, p815, MCs from SM patients		Schittenhelm et al. 2014
Curcumin	Multiple		X		HMC-1, murine BMMC	BALB/c mice	Baek et al. 2003 Kinney et al. 2015
Demethylating agents (5-azacytidine, 5-aza-2′deoxycytidine)	DNA methylation	X		(X)	HMC-1		Krug et al. 2010 Meeran et al. 2010R
EXEL-0862 (WO2004050681 A2)	KIT, STAT3	X			HMC-1		Pan et al. 2007
Fedratinib (TG101348)	JAK2 inhibition	X			HMC-1		Lasho et al. 2010
GLC756 {(3R,4aR,10aR)-l,2,3,4,4a,5,10,10a-octahydro-6-hydroxy-1-methyl-3-[(2-pyridyl-thio) methyl]-benzo [gq]uinolinehydrochloride)}	Dopamine D_1_ and D_2_ receptor agonist		X		RBL-2H3 cells		Laengle et al. 2006
Gly-Phe-CHN_2_, PZ610, PZ709, PZ889 (chemical structures not yet published)	Dipeptidylpeptidase-1 inhibitors				LAD2 MC		El-Feki et al. 2011
Histamine H_4_-receptor agonist	Histamine H_4_-receptor		X	X	HMC-1, murine MCs	Ex vivo guinea pig and murine hearts	Aldi et al. 2014
Histone deacetylase inhibitors: vorinostat, AR-42 {*N*-hydroxy-4-[[(2S)-3-methyl-2-phenylbutanoyl]amino]benzamide}	Histone deacetylase	X			HMC-1.2, primary human MCs, murine, and canine MCs		Mühlenberg et al. 2009 Hadzijusufovic et al. 2010 Meeran et al. 2010R Abdulkadir et al. 2015 Lin et al. 2010
Hypothemycin	Inhibition of KIT and Btk		X		Human MCs	Mice	Jensen et al. 2008
IMD-0354 {*N*-[3,5-bis(trifluoromethyl)phenyl]-5-chloro-2-hydroxybenzamide}	NF-κB inhibitor	X			HMC-1		Tanaka et al. 2005
JTE-052 {3-{(3R,4R)-4-methyl-3-[methyl-(7*H*-pyrrolo[2,3-d]pyrimidin-4-yl)-amino]-piperidin-1-yl}-3-oxo-propionitrile mono citrate}	JAK1,2,3 inhibitor, Tyk2 inhibitor		X		Human MCs	DBA/1J mice, Lewis rats	Tanimoto et al. 2015
Mefloquine	Permeabilization of secretory granules	X			Human and murine MCs		Paivandy et al. 2014
Mylotarg (gemtuzumab ozogamicin)	CD-33 targeting drug	X			HMC-1, human cord blood-derived MCs		Krauth et al. 2007
Neramexane	Possibly NMDA antagonist		X		HMC-1 cells		Kurzen 2009
Obatoclax	BH3 mimetic	X			HMC-1, human neoplastic BMMC		Aichberger et al. 2009
ONO-4053 (chemical structure not yet published)	Prostaglandin receptor DP1 antagonist	X			Human BMMC		Yamaguchi et al. 2016
8-OH-DPAT (7-(Dipropylamino)-5,6,7,8-tetrahydronaphthalen-1-ol)	5-HT_1A_ receptor	No effect	X				Ritter et al. 2012
Palmitoylethanolamide	PPAR-α, cannabinoid receptors, potassium channels, TRPV1			X	rat peritoneal MCs		Facci et al. 1995 Mattace Raso et al. 2014R
PD180970 {6-(2,6-dichlorophenyl)-2-(4-fluoro-3-methylanilino)-8-methylpyrido[2,3-d]pyrimidin-7-one}	KIT, Bcr-Abl, PDGFR	X			HMC-1, P815 MCs		Corbin et al. 2004
Phosphodiesterase inhibitors	Phosphodiesterase		X		Human lung MCs, rat MCs	Wistar rats	Lau and Kam 2005; Eskandari et al. 2015 Babaei and Bayat 2012
Phosphatidylethanolamine, phosphatidylserine	CD300a	X			Human cord blood-derived MCs, human lung MCs, murine BMMC		Bachelet et al. 2005 Simhadri et al. 2012
Prostaglandin D_2_ receptor antagonists	CRTH2			X			Harvima et al. 2014R
Proteases inhibitors	Tryptase, chymase, cathepsins, carboxypeptidase			X	Human and murine MCs	Mice	Caughey 2016R Harvima et al. 2014R
Rapamycin	mTOR pathway inhibitor	X			HMC-1		Chan et al. 2013
RNAi	RNA interference against *KIT* RNA	X			HMC-1		Ruano et al. 2010
Rosiglitazone, pioglitazone	PPARγ		X		Murine BMMC		Tachibana et al. 2008
Siramesine	Sigma-2 receptor agonist	X			Human and murine MCs		Spirkoski et al. 2012
Sitagliptin	Dipeptidylpeptidase-4 inhibitor		X		Rat peritoneal MCs		Nader 2011 1845
Somatostatin	Somatostatin receptors			X		Wistar rats	Tang et al. 2005
Syk kinase inhibitors	Syk kinase		X		Human, murine, and rat MCs; RBL-2H3		Matsubara et al. 2006 Finn and Walsh 2013
Tandutinib (MLN518)	KIT, STAT3	X			HMC-1, P815 MCs		Corbin et al. 2004
Tetracyclines	Multiple	X		X	Rat serosal MCs, HMC-1	Human	Sandler et al. 2005 Joks and Durkin 2011R
α-Tocopherol	Multiple	X			HMC-1		Kempna et al. 2004 Ruano et al. 2010
Tranilast	Yet to be defined		X	(X)	Rat peritoneal MC	Rats; rabbits	Adachi et al. 1999 Cooper et al. 2007 Baba et al. 2016
Whi-P131 {4-[(6,7-dimethoxyquinazolin-4-yl)amino]phenol}	JAK3/STAT pathway inhibitor	X			HMC-1		Chan et al. 2013 Bibi et al. 2014R

*R* review article (further references therein), *MC* mast cell, *BMMC* bone marrow-derived mast cells

### General considerations on drug treatment of MCAD

Although no biomarkers of symptomaticity or therapeutic response are yet validated, the tolerability and efficacy of most therapies tried in MCAD (starting, and escalating in dosage and composition, cautiously) become clinically evident within 1–2 months. Modest experiments with alternative dosages and/or dosing frequencies are not unreasonable. Therapies clearly shown clinically helpful should be continued; therapies not meeting this high bar should be halted to avoid the troublesome polypharmacy that can easily develop in such patients. With no predictors of response yet available, a cost-based approach to sequencing therapeutic trials in a given patient seems reasonable. It is not even clear yet that medications targeted at mediators found elevated in diagnostic testing (e.g., antihistamines in patients with elevated histamine, non-steroidal anti-inflammatory drugs in patients with elevated prostaglandins, leukotriene inhibitors in patients with elevated leukotrienes) are reliably effective, again perhaps unsurprising given the multitude of MC mediators and the complexity of the signaling networks dysregulated by the multiple mutations in MC regulatory elements present in most MCAD patients. Successful regimens appear highly personalized.

Multiple simultaneous (or nearly so) changes in the medication regimen are discouraged since such can confound identification of the specific therapy responsible for a given improvement (or deterioration). Ineffective or harmful agents should be stopped promptly. Prescribers should be aware that although rapid demonstration of intolerance of a new medication (or a new formulation of a previously well-tolerated medication) often suggests excipient reactivity as further discussed below, some active drug molecules themselves (e.g., cromolyn) sometimes cause an initial symptom flare which usually soon abates. Temporary waiver of gluten-, yeast-, and cow milk protein-containing foods during the initial 3–4 weeks of drug therapy can improve the response rate (Biesiekierski et al. 2011; Rodrigo et al. 2013; own unpublished experiences). When MCAD is suspected, therapies that strongly activate the immune system (e.g., vaccinations with live vaccines or autohemotherapy) must be given with caution (especially if similar therapies were previously already poorly tolerated), as such interventions sometimes dramatically worsen MCAD acutely and/or chronically.

Any drug can induce intolerance symptoms in the individual MCAD patient. In some MCAD patients, the disease creates such remarkable states of not only constitutive MC activation but also aberrant MC reactivity that such patients unfortunately experience a great propensity to react adversely to a wide variety of medication triggers. Those MCAD patients begin demonstrating (either acutely or subacutely) odd/unusual/weird/strange/bizarre/unexpected symptoms soon after beginning new medications. It is very important to note that such patients often demonstrate even a greater propensity to react to medication excipients (i.e., fillers, binders, dyes, preservatives) than to the active ingredients. When the patient tries one or more alternative formulations of a medication with the same active ingredient but sharing as few as possible (preferably none) of the excipients in the offending formulation, the patient may discover the medication to be at least tolerable and perhaps even quite effective. Furthermore, such a scenario obviously provides the patient (and physician and pharmacist) a great opportunity to identify one or more of the specific excipients which are triggering abnormal reactivity in the patient’s dysfunctional MCs, and it is those specific excipients—not the medication as a whole—that should be added to the patient’s allergy list and screened against all present medications being taken by the patient and against all future medications proposed for the patient. An MCAD patient’s physician would be wise to not assume, just because an excipient is very widely used in many medication products and appears innocuous and well tolerated in the vast majority of patients, that the same excipient will necessarily be tolerated well in MCAD patients (unpublished observation of the authors). Sometimes the specificity of the reaction is quite extraordinary. For example, patients who react to wood-based microcrystalline cellulose might tolerate cotton-based microcrystalline cellulose without any difficulty at all, or vice versa. In some cases, the pharmacist is unable to identify alternative commercially available formulations sharing few to none of the excipients in the offending formulation, and in those cases, a compounding pharmacist may need to be engaged to identify/develop a custom-compounded formulation the patient can tolerate. (There can be geographic and financial challenges in accessing compounding pharmacies, though.) Occasionally, MCAD patients may be so remarkably reactive to such a wide range of excipients that they can only tolerate a given medication when provided as pure drug salt, reconstituted in water (without preservatives). Intolerance symptoms can be mediated by IgE antibodies, though this scenario appears to be rare since the symptoms are usually not ameliorated by the anti-IgE monoclonal antibody omalizumab (unpublished observation, G.J. Molderings). Alternatively, they may be mediated by IgG antibodies, raising the question of whether gamma globulin (if itself tolerable) might be a helpful adjunct therapy in such patients (perhaps by directly targeting the MC surface’s IgG receptors or via indirect pathways). Recently, a MC-specific receptor termed MRGPRX2 has been identified which appears to be crucially involved in pseudo-allergic drug reactions (McNeil et al. 2015; Seifert 2015).

### Drugs which should not be used in MCAD

Several drugs have the ability to trigger MC mediator release. A compilation of drugs known to be associated with a high risk of release of mediators from MCs is given in Table 12. However, there often are therapeutic alternatives to these drugs (Table 12).

**Table 12:** Compilation of drugs associated with a high risk of release of mediators from mast cells and their therapeutic alternatives (compiled from Mousli et al. 1994; Sido et al. 2014; Afrin et al. 2015b; McNeil et al. 2015)

Substance group	Drugs with proven or theoretical high risk of mast cell activation	Therapeutic alternatives
Intravenous narcotics	Methohexital Phenobarbital Thiopental	Propofol Ketamine Etomidate Midazolam
Muscle relaxants	Atracurium Mivacurium Rocuronium	Cis-atracurium Vecuronium
Antibiotics	Cefuroxim Gyrase inhibitors Vancomycin	Roxithromycin
Selective dopamine- and norepinephrine reuptake inhibitors	Bupropion	Amitriptyline, doxepine, clomipramine, maprotiline
Selective serotonin reuptake inhibitors	All	
Anticonvulsive agents	Carbamazepine, topiramate	Clonazepam
Opioid analgesics	meperidine, morphine, codeine	remifentanil, alfentanil, fentanyl, oxycodon, piritramid
Peripheral-acting analgesics	Acidic non-steroidal anti-inflammatory drugs such as ASS or ibuprofen	Paracetamol, metamizol
Local anesthetics	Amide-type: lidocaine articaine Ester-type: tetracaine, procaine	prefer amide-Type, e.g., bupivacaine
Peptidergic drugs	Icatibant, cetrorelix, sermorelin, octreotide, leuprolide	
X-ray contrast medium	Iodinated contrast medium Gadolinium chelate	Non-ionic contrast media: iohexol, iopamidol, iopromida, ioxilan, ioversol, idolatran, iodixanol
Plasma substitutes	Hydroxyethyl starch Gelatine	Albumin solution, 0.9 %-NaCl solution, Ringer’s solution
Cardiovascular drugs	ACE inhibitors ß-Adrenoceptor antagonists	Sartans, calcium channel antagonists, ivabradine, and much else

## Conclusions and future perspectives

The therapeutic management of individuals with MCAD is complex and requires reviewing the entire spectrum of symptoms. The paucity of randomized, controlled studies makes treatment of refractory disease challenging and requires patience, persistence, and a methodical approach on the parts of both patient and managing provider(s). Delayed control of the symptoms may increase morbidity. Effective therapy often consists simply of antihistamines and MC-stabilizing compounds supplemented with medications targeted at specific symptoms and complications (Table 13). Current treatment options for refractory disease are based mainly on observational studies and case reports. Until larger randomized, controlled trials become available to give more guidance on therapy for refractory disease, clinicians should use the available data in conjunction with their clinical expertise and the adverse effect profile of the available drugs to make treatment decisions. More research is certainly needed to better understand MCAD pathobiology, in particular to determine which deregulated genes contribute to a specific symptom or symptom cluster. The greatest challenge in translational research for the discovery of new rational therapies requires a highly interactive interdisciplinary approach engaging basic science labs and clinicians. Understanding of the key components might hasten the progress of novel treatment for all these devastating MCAD phenotypes.

**Table 13:** Schematic summary of selected potential targets of pharmacological interventions in MCAD

Targets of drugs located in the plasma membrane	
Histamine H_1_ receptor	H_1_-antihistamines
Histamine H_2_ receptor	H_2_-antihistamines
CB1/CB2 cannabinoid receptors	Cannabinoids
cysLTR1 leukotriene receptor	CysLTR1 antagonists, e.g., montelukast
ß-Adrenoceptor	ß-Sympathomimetics
EP_2_ receptor	EP_2_ receptor agonist, e.g., butaprost
Chemokine receptors	Chemokines
FcεRI	IgE antibody, e.g., omalizumab
FcγRIII	IgG
Siglec-8	Siglec-8 ligand
CD300a	Phosphatidylethanolamine, phosphatidylserine
Targetting released mast cell mediators	
Tryptase	Tryptase inhibitor, e.g., nafamostat
Chymase	Chymase inhibitor, e.g., BCEAB (4-[1-[bis-(4-methyl-pheny)-methyl]-3-(2-ethoxy-benzyl)-4-oxo-azetidine-2-yloxy]-benzoic acid)
Cathepsin G	Cathepsin G inhibitor, e.g., RWJ355871 (β-ketophosphonate 1)
TNFα	Infliximab, adalimumab
IL-4	Pascolizumab
IL-5	e.g., mepolizumab
IL-6	e.g., sirukumab
IL-17	e.g., secukinumab
Intracellular inhibition of mediator formation	
Histamine	Histidine decarboxylase inhibition, e.g., by vitamin C
Leukotrienes	5-Lipoxygenase inhibitors, e.g., zileuton
Prostaglandins	Cyclooxygenase inhibitors, e.g., acetylsalicylic acid, etoricoxib
Inhibition of cytosolic pathways	
Signaling pathways containing protein kinases	Inhibitors of protein kinases (see Table 8)
mTOR pathway	e.g., rapamycin, everolimus
Apoptotic pathways	Stimulation of apoptosis by, e.g., ABT-737, obatoclax
Intranuclear targets	
Histone deacetylase	Histone deacetylase inhibitors, e.g., vorinostat
DNA methylation	Demethylating agents, e.g., 5-azacytidine, 5-aza-2′deoxycytidine
DNA	Nucleoside analog cladribine
